# Subphenotypic features of patients with sepsis and ARDS: a multicenter cohort study

**DOI:** 10.3389/fmed.2024.1476512

**Published:** 2024-11-01

**Authors:** Nan Li, DeYu Fang, Feng Ge, Lin Zhang, Ying Liu, Hongxu Jin, Hao Shen, Keliang Xie, Yan Gao

**Affiliations:** ^1^Department of Emergency Medicine, General Hospital of Northern Theater Command, Shenyang, China; ^2^Department of Chemistry, Liaoning University of Traditional Chinese Medicine, Shenyang, Liaoning, China; ^3^Department of Biochemistry and Molecular Biology, Liaoning University of Traditional Chinese Medicine, Shenyang, Liaoning, China; ^4^Department of Critical Care Medicine, Tianjin Beichen Hospital, Tianjin, China; ^5^Department of Critical Care Medicine, Tianjin Medical University General Hospital, Tianjin, China; ^6^Department of Anesthesiology, Tianjin Institute of Anesthesiology, Tianjin Medical University General Hospital, Tianjin, China

**Keywords:** sepsis, acute respiratory distress syndrome (ARDS), pulmonary infections, non-pulmonary infections, subphenotypic

## Abstract

**Objectives:**

Patients with sepsis are often comorbid with acute respiratory distress syndrome (ARDS), and the phenotypic characteristics of pulmonary and non-pulmonary infections leading to ARDS are still unclear. This study aimed to compare the phenotypic characteristics of ARDS resulting from pulmonary infections and other non-site infections and provide better guidance for clinical treatment.

**Methods:**

We conducted a multicenter cohort analysis using data from the Tianjin Medical University General Hospital, Medical Information Mart for Intensive Care-IV (MIMIC-IV), and the electronic intensive care unit (eICU) databases. The study population consisted of adult patients diagnosed with sepsis and ARDS. The primary objectives were to compare the characteristics and outcomes of patients with pulmonary infection-induced ARDS and those with non-pulmonary infection-induced ARDS using Wilcoxon analysis, Kaplan–Meier curves, correlation analysis, propensity matching scores, and other statistical methods.

**Results:**

Patients with ARDS by pulmonary infection may be more likely to have a history of chronic obstructive pulmonary disease, and abdominal infection was more likely to induce ARDS in sepsis patients with non-pulmonary infection. Pulmonary infections caused by *Klebsiella pneumoniae* and *Acinetobacter baumannii* were more likely to induce ARDS. The oxygenation index and prognosis of ARDS patients induced by pulmonary infection were worse than those caused by other infections, with lower PaO_2_, PaO_2_/FiO_2_, and ROX index and longer hospital stay. More ARDS patients with pulmonary infection were given mechanical ventilation therapy, with higher mortality, APACHE II, SOFA, and SAPS II. The further correlation analysis showed that the prognostic scores of ARDS patients were negatively correlated with PaO_2_/FiO_2_ and ROX index. The above results were confirmed to varying degrees by propensity matching scores, external cohort validation, and other methods.

**Conclusion:**

Pulmonary infection induces a worse prognosis of ARDS than other site infections in patients with sepsis and ARDS. These patients require heightened vigilance, early intervention, and possibly more aggressive management strategies.

## 1 Introduction

Acute respiratory distress syndrome (ARDS) is a diffuse lung injury caused by intrapulmonary and extrapulmonary factors over a short period of time and is histologically characterized by diffuse alveolar injury, including pulmonary edema, hyaline membrane formation, alveolar hemorrhage, and inflammation (1, 2). Characterized by non-cardiogenic pulmonary edema and profound hypoxemia, ARDS poses a significant challenge in intensive care units worldwide, with mild, moderate, and severe cases of ARDS having mortality rates of 34.9, 40.3, and 46.1%, respectively (3). Despite advances in supportive care, sepsis and ARDS remain the main cause of sepsis (4). Patients with ARDS still have a poor prognosis. ARDS is a heterogeneous syndrome, and the disease characteristics and prognosis of patients with ARDS vary depending on the cause (5). Therefore, it is particularly important to study the characteristics of ARDS disease caused by different causes and improve the prognosis of ARDS patients.

Sepsis is the most predominant extrapulmonary cause of ARDS, accounting for approximately 32% of ARDS cases (6). Several studies have shown that sepsis-induced ARDS tends to be more severe than other factors, resulting in a worse prognosis and higher mortality (7, 8). A recent study found significant differences in metabolic patterns between patients with sepsis-induced ARDS and non-ARDS controls and between sepsis-induced direct and indirect ARDS subtypes (9). As ARDS is a heterogeneous disease in terms of etiology and clinical aspects, based on the above findings, we consider that the disease characteristics and prognosis of ARDS induced by the different sites of infection in sepsis are different. In patients with sepsis, the ARDS subphenotype is usually divided into direct (pulmonary) ARDS and indirect (extrapulmonary) ARDS based on the source of infection. We hypothesize that in patients with sepsis, pulmonary infections with ARDS may differ in disease features and prognosis from those due to other site infections.

At present, the subphenotypic characteristics of patients with pulmonary infection and extrapulmonary infection-induced ARDS in sepsis remain unclear, and large cohort studies are lacking. Our primary objective was to elucidate the unique features and outcomes of sepsis-induced ARDS based on pulmonary infections and extrapulmonary infections and support the development of personalized approaches to critical care management in sepsis patients and ARDS.

## 2 Materials and methods

### 2.1 Data source

This cohort study was based on the Tianjin Medical University General Hospital from 2019 and 2024, MIMIC-IV database (version 2.2) from 2008 and 2019, and the eICU-CRD database (version 2.0) from 2014 and 2015 (No.0403000206). Tianjin Medical University General Hospital was approved by the hospital’s Ethics Committee (IRB2022-YX-041-01). The MIMIC-IV and eICU-CRD were approved by the Institutional Review Board of the Beth Israel Deaconess Medical Center (2001-P001699/14) and the Massachusetts Institute of Technology (No. 0403000206). The requirement for informed consent was waived because the data of all patients in the database were anonymized (10, 11).

### 2.2 Study population and data extraction

We included all patients who were first admitted to the ICU with ARDS from the Tianjin Medical University General Hospital, the MIMIC-IV database, and the eICU-CRD database. To extract the raw data from the three cohort studies, the patients met the following criteria: They were diagnosed with sepsis 3.0 (12) and ARDS according to the Berlin criteria (13). In addition, ARDS patients had a concomitant at the site of infection according to the ICD diagnostic code. Abdominal infections include acute cholecystitis, acute appendicitis, peritonitis, intra-abdominal abscess, liver abscess, periappendiceal abscess, gastroduodenal perforation, pancreatic abscess, and other diseases. We excluded the following: (i) patients younger than 18 years old; (ii) ICU stay of less than 24 h; (iii) the patients who were diagnosed with congestive heart failure, cardiogenic pulmonary edema, extensive atelectasis, alveolar hemorrhage, massive pleural effusion, pulmonary hypertension, and interstitial pulmonary disease; and (iv) patients with missing values of oxygenation-related indicators.

The extracted data included demographics, comorbidities, site of infections, pathogenic microorganisms, and respiratory-related indicators. The following demographic information was extracted: age, sex, and length of ICU stay. Data regarding comorbidities including hypertension, diabetes, chronic obstructive pulmonary disease (COPD), cardiovascular, hepatic disease, and chronic kidney disease were extracted. Respiratory-related indicators such as respiratory rate (RR), oxygenated hemoglobin saturation (SpO_2_), partial pressure of oxygen (PaO_2_), partial pressure of carbon dioxide (PaCO_2_), a fraction of inspired oxygen (FiO_2_), ROX, and PEEP (positive end-expiratory pressure) were collected. We used the median values of SpO_2_, PaO_2_, FiO_2_, RR, and PEEP measurements during oxygen therapy as a measure of the central tendency of oxygen exposure. In addition, we extracted details of the patient who underwent mechanical ventilation, the use of inotropic/vasopressor support, and renal replacement therapy. The Sequential Organ Failure Assessment (SOFA), Acute Physiology and Chronic Health Evaluation (APACHE II) scores, and Simplified Acute Physiology Score II (SAPS II) scores, which represent the severity of the disease, were also included. Although the ROX index is used to assess the need for endotracheal intubation in patients receiving high-flow oxygen, this study included endotracheal intubation patients to observe whether the ROX index is meaningful in this study and whether it has a suggestive effect on patients with pulmonary and non-pulmonary infections. The raw data were extracted using a structure query language (SQL) with Navicat and further processed with R software.

### 2.3 Statistical analysis

Patient characteristics were described overall and by group (non-pulmonary infections and pulmonary infections). The data were analyzed using the R software. Data distributions were analyzed by the Shapiro–Wilk test. All the data exhibited skewed distributions. Continuous data (age, PaCO_2_, FiO_2_, PaO_2_, SpO_2_, the length of ICU stay, SOFA, APACHE II, and SAPS II scores) were expressed as median and interquartile range (IQR). The other categorical data were expressed in counts and proportions. The continuous variables were examined using the non-parametric Mann–Whitney U-test. Furthermore, categorical variables were compared using the Fisher exact test. Propensity matching score was used to adjust for confounders between the non-pulmonary infections and pulmonary infections groups to verify the reliability of the results of the study. Supplementary material 3 shows the comparison of baseline data after propensity matching between sepsis patients and ARDS combined with pulmonary infection and sepsis patients and ARDS combined with non-pulmonary infection. The standardized mean difference (SMD) was used to assess the quality of the propensity score matching. If the SMD does not exceed 0.1, the matching quality for this variable is generally considered acceptable. Supplementary material 4 showed that the matching effect of patients in the two groups was better. Kaplan–Meier curves were used to evaluate the prognosis of sepsis patients and ARDS in the two groups of Tianjin Medical University General Hospital non-propensity matching and propensity matching and the prognosis of two groups of patients in the two externally validated cohorts. In this study, patients with missing values of more than 20% were removed, and the method of multiple imputation was used to deal with the missing values. The R software package^1^ was used to perform all statistical analyses. Statistical differences were considered significant at a *p*-value of <0.05.

## 3 Results

### 3.1 Baseline characteristics

A total of 11,823 patients with sepsis met the ARDS Berlin diagnostic criteria from Tianjin Medical University General Hospital, MIMIC-IV, and the eICU databases. A total of 9,098 patients (congestive heart failure and cardiogenic pulmonary edema (*n* = 5,507), large pleural effusion and alveolar hemorrhage (*n* = 45), massive pleural effusion (*n* = 137), age less than 18 years (*n* = 6), missing blood oxygen-related indexes (*n* = 1,210), hospital stay of less than 24 h (*n* = 1730), and missing data of more than 20% (*n* = 463) were excluded based on the exclusion criteria. A total of 2,725 patients were included in the study. The number of patients with pulmonary infections and ARDS was 848, whereas the number of patients with non-pulmonary infections and ARDS was 1877 in three cohorts.

### 3.2 Patients with pulmonary infections and ARDS have worse oxygenation indicators

Table 1 and Supplementary material 1 summarize the characteristics of the sepsis patients with ARDS in Tianjin Medical University General Hospital. The median age of the patients was 67 years. Before matching, patients with a pre-existing history of COPD were more likely to develop pulmonary infections combined with ARDS (43.2% vs. 16.1%, *p* < 0.001), who were more likely to be infected with *Acinetobacter baumannii* and *Klebsiella pneumoniae*. Pulmonary infections combined with ARDS had worse respiratory-related indicators, including higher respiratory rate, lower SpO_2_, PaO_2_, SpO_2_/FiO_2,_ and PaO_2_/FiO_2_, and need for higher respiratory support parameters (FiO_2_) (*p* < 0.001) (Table 1). An external cohort study of the eICU database showed that sepsis patients with pulmonary infections and ARDS had a lower ROX (*p* = 0.003) and PaO_2_/FiO_2_ (*p* = 0.009), and the above findings were confirmed by an external cohort study of the eICU database (Supplementary materials 4, 5).

**Table 1:** Baseline data of sepsis patients and ARDS in Tianjin Medical University General Hospital.

	Original cohort			Match cohort		
Characteristic	Non-pulmonary infections and ARDS (*n* = 391)	Pulmonary infections and ARDS (*n* = 405)	*p*	Non-pulmonary infections and ARDS (*n* = 387)	Pulmonary infections and ARDS (*n* = 38 7) *p*	*p*
Age, years	67.00 [57.00, 75.00]	67.00 [55.00, 75.00]	0.927	67.00 [57.00, 75.00]	67.00 [55.00, 74.00]	0.52
Male sex, *n* (%)	154 (39.4)	138 (34.1)	0.139	153 (39.5)	120 (31.0)	0.016
Co-morbid conditions, *n* (%)						
Hypertension	214 (54.7)	218 (53.8)	0.853	212 (54.8)	213 (55.0)	1
Diabetes	123 (31.5)	118 (29.1)	0.525	121 (31.3)	99 (25.6)	0.094
Chronic obstructive pulmonary disease	63 (16.1)	175 (43.2)	<0.001	135 (34.9)	120 (31.0)	0.284
Cardiovascular	137 (35.0)	115 (28.4)	0.053	135 (34.9)	120 (31.0)	0.284
Hepatic	55 (14.1)	42 (10.4)	0.137	55 (14.2)	30 (7.8)	0.006
Chronic kidney disease	94 (24.0)	80 (19.8)	0.168	94 (24.3)	48 (12.4)	<0.001
Site of infection, *n* (%)						
pulmonary	0 (0.0)	405 (100.0)	<0.001	0 (0.0)	387 (100.0)	<0.001
Abdominal	165 (42.2)	0 (0.0)	<0.001	164 (42.4)	0 (0.0)	<0.001
Urinary	76 (19.4)	0 (0.0)	<0.001	75 (19.4)	0 (0.0)	<0.001
Skin soft tissue	70 (17.9)	0 (0.0)	<0.001	68 (17.6)	0 (0.0)	<0.001
Catheter	41 (10.5)	0 (0.0)	<0.001	41 (10.6)	0 (0.0)	<0.001
others	55 (14.1)	0 (0.0)	<0.001	51(13.2)	0 (0.0)	<0.010
Pathogenic microorganisms, *n* (%)						
*Acinetobacter baumannii*	56 (14.3)	105 (25.9)	<0.001	55 (14.2)	48 (12.4)	0.525
*Klebsiella pneumoniae*	74 (18.9)	127 (31.4)	<0.001	72 (18.6)	54 (14.0)	0.098
*Pseudomonas aeruginosa*	42 (10.7)	52 (12.8)	0.420	41 (10.6)	57 (14.7)	0.105
Escherichiacoli	35 (9.0)	28 (6.9)	0.351	35 (9.0)	15 (3.9)	0.005
*Staphylococcus aureus*	33 (8.4)	34 (8.4)	1.000	41 (10.6)	48 (12.4)	0.499
Respiration						
Respiratory rate, breaths per minute	23.00 [18.00, 29.00]	25.00 [20.00, 32.00]	0.001	23.00 [18.00, 29.00]	24.00 [18.00, 31.00]	0.105
SpO_2_, %	96.00 [94.00, 98.00]	93.00 [92.00, 95.00]	<0.001	95.00 [92.00, 97.00]	94.00 [90.00, 97.00]	0.031
PaO_2,_ mmHg; median	109.40 [88.59, 134.12]	92.00 [73.75, 120.09]	<0.001	106.19 [84.85, 136.75]	89.90 [72.50, 128.90]	<0.001
PaCO_2_, mmHg	38.00 [32.00, 44.00]	37.00 [32.10, 44.90]	0.987	38.00 [32.00, 44.00]	37.00 [32.20, 41.30]	0.496
FiO_2_	0.53 [0.50, 0.80]	0.61 [0.50, 1.00]	<0.001	0.53 [0.50, 0.80]	0.60 [0.50, 1.00]	0.118
PaO_2_/FiO_2_ ratio, mmHg	194.39 [139.00, 238.81]	142.83 [88.75, 207.84]	<0.001	194.60 [139.00, 239.19]	158.20 [110.00, 219.10]	<0.001
SpO_2_/FiO_2_ ratio	165.00 [114.38, 198.00]	142.00[93.00, 190.00]	<0.001	165.00 [114.38, 198.00]	157.38 [96.00, 198.00]	0.186
ROX	6.69 [4.66, 9.73]	5.62 [3.87, 8.00]	<0.001	6.69 [4.66, 9.73]	6.15 [4.48, 8.73]	0.03
PEEP, cmH_2_O	7.00 [6.00, 8.00]	7.00 [6.00, 8.00]	0.29	7.00 [6.00, 8.00]	7.00 [6.00, 8.00]	0.299

SpO_2_, oxygen saturation; PaO_2_, partial pressure of oxygen in the arterial blood; FiO_2_, fraction of inspiration O_2_.

The above results suggest that compared to other site infections and ARDS, patients with pulmonary infection and ARDS had worse oxygenation indexes; to verify the accuracy of the study results, we used PSM to balance the baseline characteristics (Supplementary material 2). We matched nine variables, including a history of COPD, infection with *Klebsiella pneumoniae* and *Acinetobacter baumannii,* and others (Supplementary material 3). After matching, the study results suggested that patients with pulmonary infections and ARDS also had worse indicators of oxygenation (Table 1).

### 3.3 Outcomes

#### 3.3.1 Patients with pulmonary infections and ARDS had longer hospital stays and periods of mechanical ventilation

We compared the prognosis of patients with pulmonary infection with ARDS and patients with non-pulmonary infection with ARDS in the Tianjin Medical University General Hospital Cohort. The patients with pulmonary infections with ARDS had a worse prognosis, including longer hospital stays (*p* = 0.014), duration of mechanical ventilation (*p* < 0.001), and more patients given renal replacement therapy (*p* = 0.002). After matching, we still confirmed that sepsis patients with pulmonary infections and ARDS had a poorer prognosis, including longer hospital stays, longer periods of mechanical ventilation, and more patients requiring treatment with vasoactive or positive inotropic drug therapy (Table 2). The above results were validated by external MIMIC-IV and eICU cohorts to varying degrees (Supplementary materials 4, 5).

**Table 2:** The outcome of sepsis patients and ARDS.

Original cohort				Match cohort		
Characteristic	Non-pulmonary infections and ARDS (*n* = 391)	Pulmonary infections and ARDS (*n* = 405)	*p*	Non-pulmonary infections and ARDS (*n* = 391)	Pulmonary infections and ARDS (*n* = 405)	*p*
ICU length of stay (days)	11.00 [5.00,21.00]	13.41 [7.00, 24.00]	0.014	11.00 [5.00, 20.95]	14.00 [5.00, 25.00]	0.046
Inotropic/vasopressor support, *n* (%)	242 (61.9)	262 (64.7)	0.456	240 (62.0)	201 (51.9)	0.006
Ventilator-free (Ventilator -support) days	6.18 [2.69, 13.59]	9.85 [4.00, 16.19]	<0.01	6.18 [2.69, 13.25]	8.83 [3.46, 17.00]	<0.001
Renal replacement therapy, *n* (%)	119 (30.4)	168 (41.5)	0.002	119 (30.7)	129 (33.3)	0.488

#### 3.3.2 Patients with pulmonary infections and ARDS had more severe disease severity

**Figure 1:**
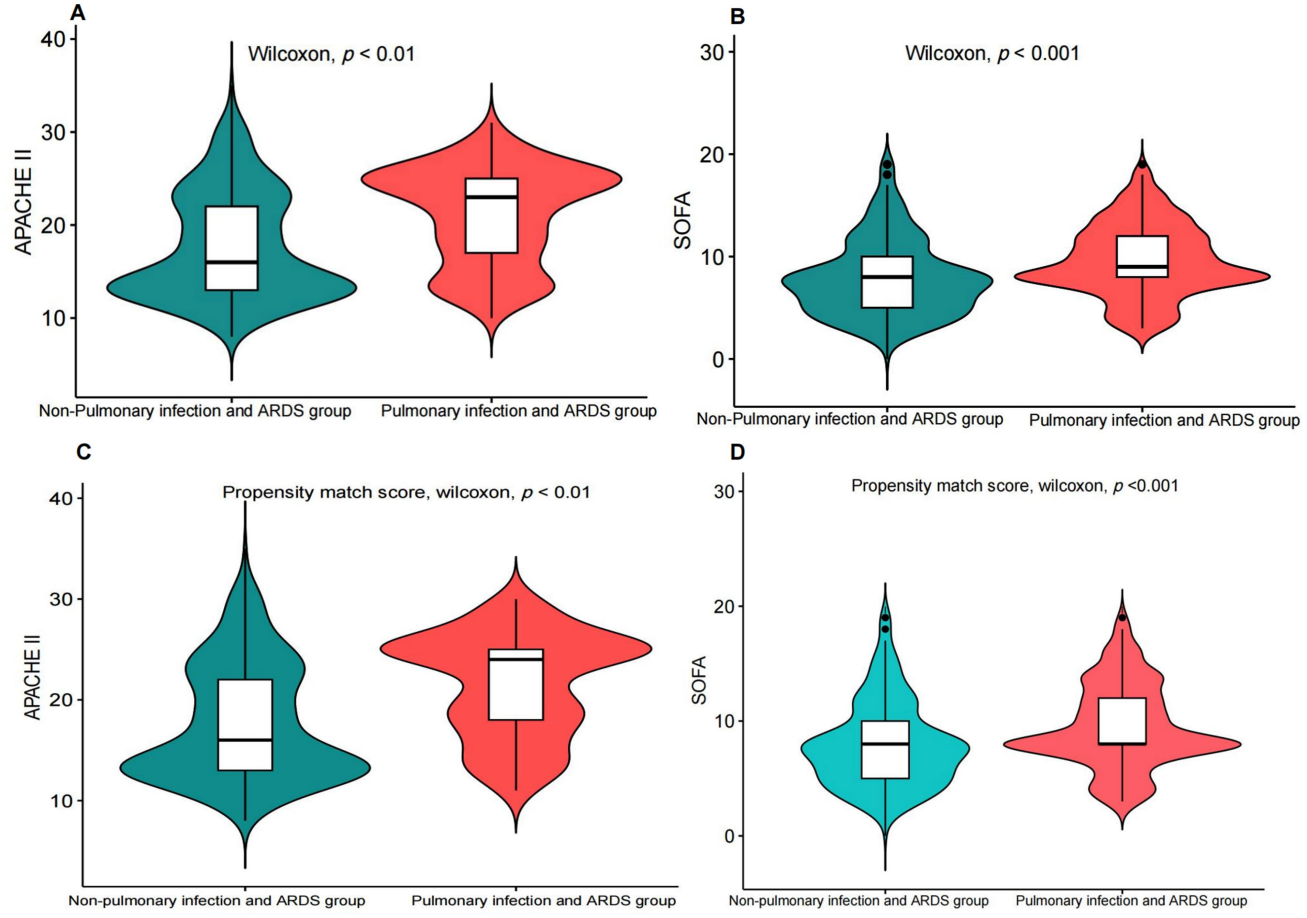
Comparison of the SOFA score and the APACHE II score between patients with pulmonary infectionwith ARDS and non-pulmonary infection with ARDS. **(A,B)** Before propensity matching, comparison of the SOFA score and the APACHE II score between patients with pulmonary infectionwith ARDS and non-pulmonary infection with ARDS. **(C,D)** After propensity matching, comparison of the SOFA score and the APACHE II score between patients with pulmonary infection and ARDS and non-pulmonary infection and ARDS.

**Figure 2:**
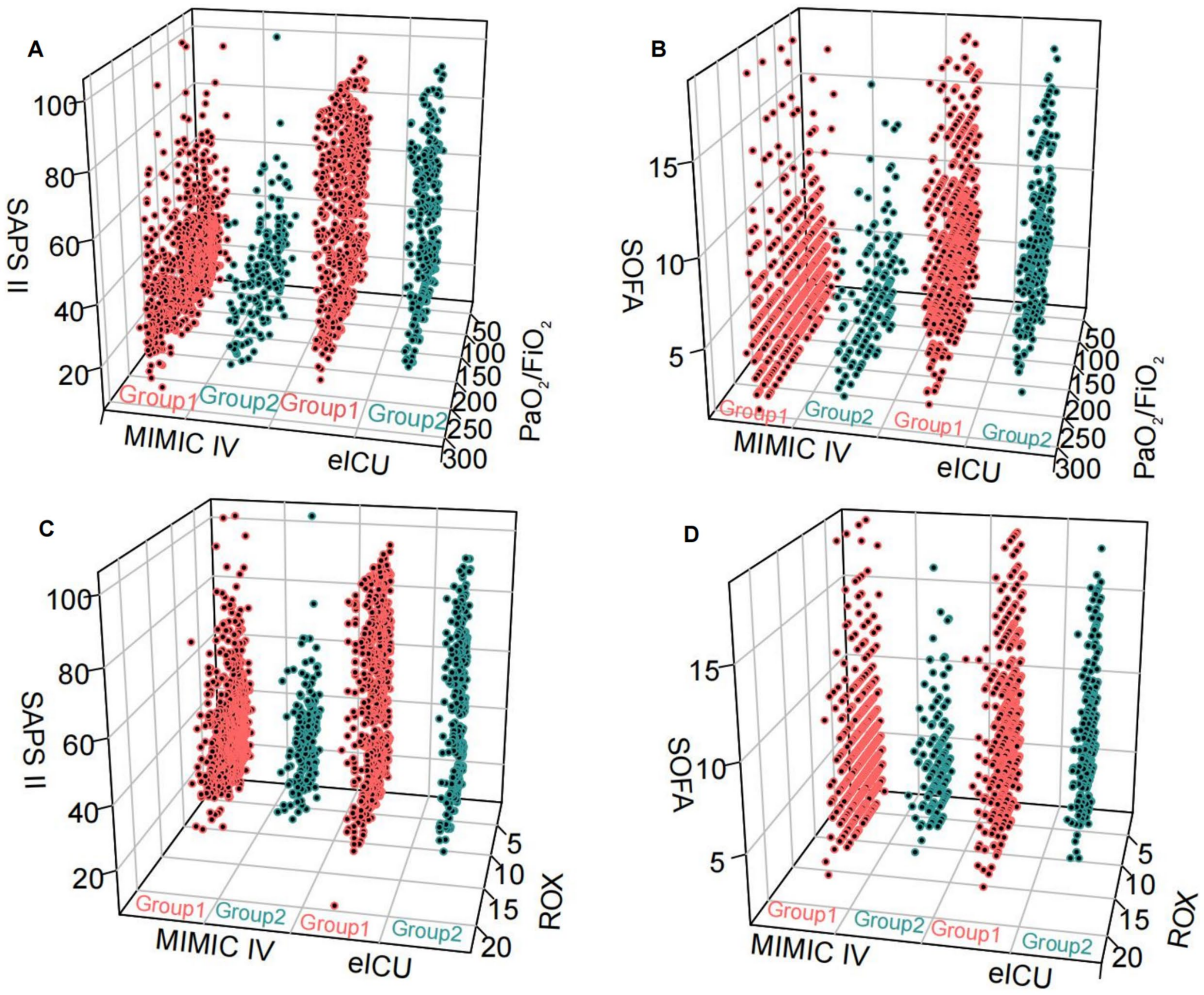
The relationship between the two groups and SOFA, SASP II score, PaO2/FiO2 and ROX index was tested in an external database. Group 1: pulmonary infection and ARDS; Group 2: non-pulmonary infection and ARDS. **(A,B)** pulmonary infection and ARDS and non-pulmonary infection and ARDS compared with SOFA, SAPS II scores, and PaO2/FiO2 levels in MIMIC IV and eICU databases. **(C,D)** Pulmonary infection and ARDS and non-pulmonary infection and ARDS compared with SOFA, SAPS II scores, and ROX levels in MIMIC IV and eICU databases.

Compared to patients with non-pulmonary infection and ARDS, the patients with pulmonary infections and ARDS had higher SOFA and APACHE II scores (Figures 1A,B) in the Tianjin Medical University General Hospital Cohort. The findings were further confirmed by the results of the propensity-matched score study (Figures 1C,D). The results of the externally validated eICU cohort were consistent with the Tianjin Medical University General Hospital cohort; however, the above findings in the above two cohorts were not confirmed in the MIMIC-IV database (Figure 2; Supplementary material 6).

#### 3.3.3 Patients with pulmonary infections and ARDS had higher 28-day mortality

**Figure 3:**
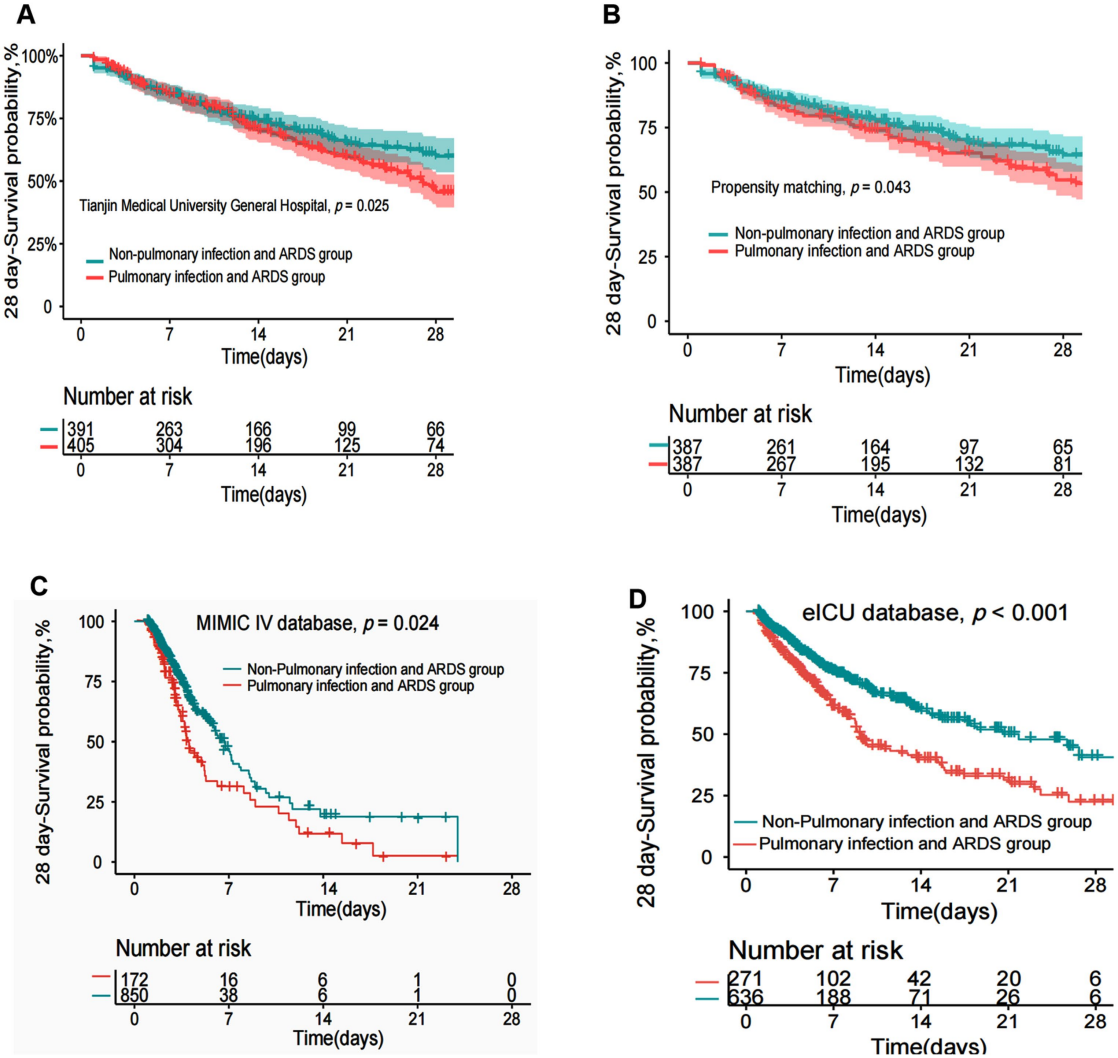
KM curves of 28-day mortality in patients with pulmonary infection and ARDS and non-pulmonary infection and ARDS. **(A)** KM curves of 28-day mortality in patients with pulmonary infection and ARDS and non-pulmonary infection and ARDS in Tianjin Medical University General Hospital. **(B)** After the propensity match score, KM curves of 28-day mortality in patients with pulmonary infection and ARDS and non-pulmonary infection and ARDS in Tianjin Medical University General Hospital. **(C,D)** KM curves of 28-day mortality in patients with pulmonary infection and ARDS and non-pulmonary infection and ARDS in MIMIC VI database and eICU database.

Our study found that the 28-day mortality rate was significantly higher in sepsis patients with pulmonary infection and ARDS than in sepsis patients with other site infection and ARDS before and after the propensity-matched score (Figures 3A,B). The above findings were confirmed by external databases, MIMIC-IV, and eICU cohorts (Figures 3C,D). However, we found no difference in 90-day mortality between sepsis patients with pulmonary infections and ARDS and non-pulmonary infections with ARDS, including after propensity matching scores in the cohort of Tianjin Medical University General Hospital (Supplementary material 7).

### 3.4 Correlation analysis of PaO_2_/FiO_2_ and ROX index with SOFA, APACHE II, and SAPS II scores

**Figure 4:**
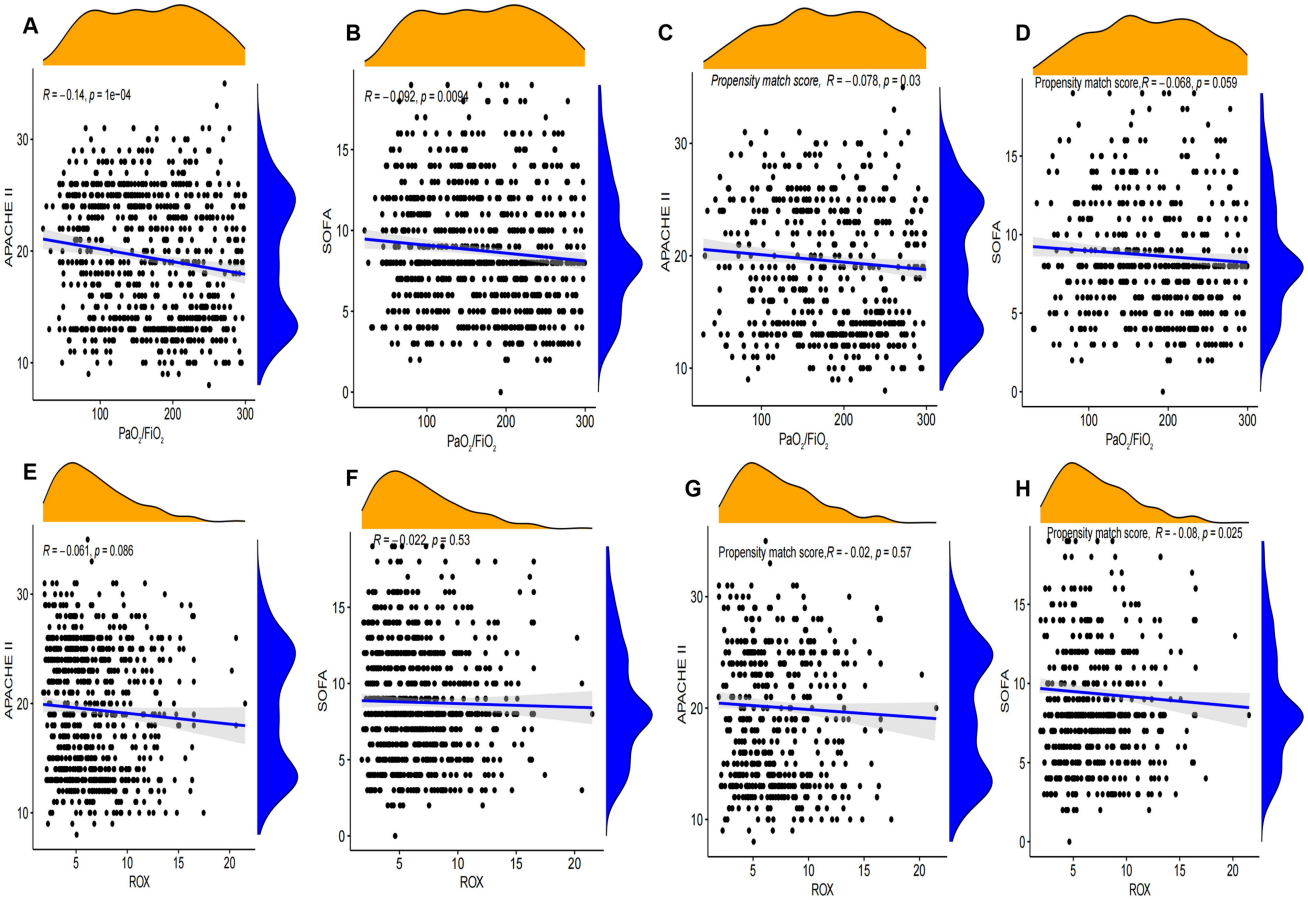
Correlation analysis of SOFA and APACHE II with SpO_2_/FiO_2_ and ROX index. **(A–D)** Correlation analysis between SOFA and APACHE II scores and PaO_2_/FiO_2_ before and after propensity matching scores in Tianjin Medical University General Hospital. **(E–H)** Correlation analysis between SOFA and APACHE II scores and ROX before and after propensity matching scores in Tianjin Medical University General Hospital.

The results of the propensity matching score study suggested that the PaO_2_/FiO_2_ and ROX appear to be a significantly negative correlation with APACHE II and SOFA scores in the General Hospital Data (Figure 4). We further used external databases to validate the relationship among PaO_2_/FiO_2_, ROX, SOFA, and SAPS II scores. External validation study findings suggested that SOFA score was negatively correlated with SpO_2_/FiO_2_ and ROX index in the eICU database. However, the results of the MIMIC-IV database were not confirmed it (Supplementary material 8).

## 4 Discussion

The present study aimed to elucidate the differences in characteristics and outcomes between sepsis patients and ARDS due to pulmonary infections and extrapulmonary infections. Our comprehensive analysis revealed several crucial findings that had important implications for the management of ARDS in the context of sepsis: Patients with a COPD disease and infected with *Klebsiella pneumoniae* and *Acinetobacter baumannii* were more likely to develop ARDS; sepsis patients and ARDS induced by pulmonary infection had worse oxygenation and worse prognosis than sepsis patients and ARDS induced by extrapulmonary infection.

The study highlighted that patients with pre-existing conditions such as COPD and infected with *Klebsiella pneumoniae* and *Acinetobacter baumannii* were more susceptible to developing ARDS in the context of pulmonary infections. COPD is one of the most common comorbidities in ARDS, patients with COPD were more likely to develop ARDS based on pulmonary infection, and our findings are consistent with previous studies (14). Drug-resistant *Klebsiella pneumoniae* and *Acinetobacter baumannii* are the main pathogens of poor prognosis of pulmonary infection, and they are also the difficulties that we have been paying attention to and overcoming (15); this study confirms that they were also important risk factors for the development of ARDS in pulmonary infections. These patients not only face higher risks of developing severe ARDS but also have poorer respiratory function and require more intensive respiratory support (16). This underscores the need for proactive monitoring and early intervention strategies in this high-risk subgroup (9).

Our results indicate that sepsis patients with pulmonary infections and ARDS had significantly worse clinical outcomes than those with ARDS from non-pulmonary infections. Sepsis patients with pulmonary infections and ARDS exhibited higher SOFA and APACHE II scores and longer ICU stays; these findings persisted even after propensity matching score, reinforcing the robustness of our observations. Pulmonary infections and ARDS had greater disease severity; this suggests that special attention and possibly more aggressive therapeutic interventions might be required for sepsis patients developing ARDS due to pulmonary infections, and the poor prognosis of ARDS and pulmonary infection may be related to the lack of early and accurate diagnosis methods and optimized treatment options. Alterations in the early pulmonary microbiota significantly increase bacterial load and biofilm formation, which further leads to a deterioration of the condition of the pulmonary; therefore, for people with ARDS, the prognosis for patients with pulmonary infections is worse (17–19). Prolonged hospitalization and mechanical ventilation need for intensive care resources indicate a higher burden on healthcare systems and suggest the necessity for efficient resource allocation and management strategies in ICUs to handle such complex cases effectively.

Sepsis patients and ARDS induced by pulmonary infection showed worse oxygenation index and higher mortality. In clinical practice, the incidence of sepsis or ARDS is higher than that of sepsis-induced ARDS, but the prognosis of sepsis-induced ARDS is worse (20). Sepsis-associated ARDS had a lower PaO_2_/FiO_2_ ratio, more pronounced dyspnea, longer recovery time, and lower extubation success rates compared with non-sepsis-associated ARDS (7). Therefore, sepsis-induced ARDS is a noteworthy group, and further study of this subgroup may greatly reduce mortality from respiratory causes in the ICU. However, further subtype analysis of patients with sepsis-induced ARDS found that the prognosis and oxygenation indicators of patients with pulmonary infection and ARDS were worse than that of patients with infection at other sites, which may be attributed to pulmonary factors differing from external pulmonary factors in the pathophysiological mechanisms that contribute to the development of ARDS (4). Therefore, we need to closely monitor pulmonary infection and ARDS in patients with sepsis; more aggressive treatment includes medication, respiratory management, and even ECMO as mortality is higher in these patients. Future research should focus on prospective studies to confirm these findings and explore targeted interventions for sepsis patients and ARDS due to pulmonary infections. Investigating the underlying mechanisms driving the worse outcomes in this subgroup could also provide insights for developing novel therapeutic strategies. Furthermore, there is a need for studies exploring the role of early and aggressive management protocols tailored specifically for high-risk patients with pre-existing conditions such as COPD.

Our correlation analysis showed a significant relationship between oxygenation indices (such as PaO_2_/FiO_2_ and ROX index) and severity scores (SOFA, APACHE II, and SAPS II). Lower oxygenation indices were associated with higher severity scores, indicating worse patient outcomes (8, 21). This reinforces the importance of these indices as critical markers in the early identification and ongoing assessment of ARDS severity in sepsis patients (22).

This study has several limitations. The retrospective nature of the analysis may introduce selection bias, and while propensity score matching was used to minimize confounders, residual confounding cannot be entirely excluded. In addition, the databases used, while comprehensive, may not capture all potential variables influencing patient outcomes, such as specific treatment modalities and their timing. The consistency of our findings across the multiple study cohorts strengthens the validity of our results. The external validation supports the generalizability of our conclusion to broader ICU populations beyond the initial study cohort. This external validation is a significant strength of our study, offering confidence in the reliability of our data and the applicability of our findings in diverse clinical settings.

## 5 Conclusion

Our study highlights that sepsis patients with pulmonary infections and ARDS had significantly worse outcomes than those with ARDS from extrapulmonary infections. These findings underscore the need for heightened vigilance, early intervention, and potentially more aggressive management strategies for this vulnerable patient population. Enhanced understanding and stratification of ARDS in the context of sepsis can lead to improved patient outcomes and more efficient utilization of critical care resources.

## Data Availability

Publicly available datasets were analyzed in this study. This data can be found here: If the reason is reasonable, the original data can be requested from the corresponding author.

## Footnotes

^1^http://www.R-project.org, The R Foundation.
