# Supplementary material for: Subphenotypic features of patients with sepsis and ARDS: a multicenter cohort study
Source: Front Med (Lausanne). 2024 Nov 1;11:1476512. doi: 10.3389/fmed.2024.1476512 (PMC11563817; doi:10.3389/fmed.2024.1476512)
Supplement: Supplementary file 1 [file Data_Sheet_1.docx]

| **Supplementary Material 1** Baseline data of sepsis patients and ARDS in Tianjin Medical University General Hospital | | | |
| --- | --- | --- | --- |
| **Characteristic** | **Non-pulmonary infections and ARDS(n=391)** | **Pulmonary infections and ARDS(n=405)** | ***P*** |
| **Indicators of infection** | | | |
| WBCx10ˆ9/L | 13.89 [9.07, 21.13] | 13.74 [9.75, 19.21] | 0.927 |
| CRP(mg/L) | 16.70 [6.86, 20.00] | 9.76 [4.06, 20.00] | <0.001 |
| PCT(ng/mL) | 2.99 [0.92, 10.76] | 2.24 [0.75, 7.41] | 0.059 |
| **Physiology** | | | |
| Temperature,°C | 37.00 [36.50, 38.30] | 37.40 [36.50, 38.50] | 0.188 |
| Heart rate, beats per minute | 103.00 [87.00, 120.50] | 110.00 [92.00, 127.00] | 0.003 |
| Systolicblood pressure, mmHg | 115.00 [96.00, 135.00] | 115.00 [91.00, 143.00] | 0.768 |
| Diastolicblood pressure, mmHg | 60.00 [49.00, 75.00] | 60.00 [48.00, 74.00] | 0.699 |
| **Laboratory indicators** | | | |
| Platelet (×10ˆ9 /L) | 124.00 [63.00, 190.00] | 123.00 [48.00, 196.00] | 0.563 |
| Hemoglobin(g/dL) | 82.00 [66.00, 105.50] | 82.00 [67.00, 109.00] | 0.229 |
| PT(sec) | 13.20 [11.80, 15.00] | 13.40 [12.20, 15.40] | 0.047 |
| INR | 1.22 [1.09, 1.38] | 1.24 [1.12, 1.41] | 0.083 |
| APTT(sec) | 31.00 [28.10, 34.40] | 31.40 [27.90, 37.10] | 0.072 |
| ALT(U/L) | 30.00 [17.00, 62.00] | 35.00 [21.00, 75.00] | 0.037 |
| AST(U/L) | 44.00 [29.00, 102.00] | 55.00 [34.00, 108.00] | 0.010 |
| Albumin(g/L) | 31.00 [29.00, 33.00] | 31.00 [27.00, 33.00] | 0.046 |
| Creatinine(umol/L) | 163.00 [76.00, 267.00] | 150.00 [80.00, 298.00] | 0.753 |
| Bun (mmol/L) | 15.00 [9.00, 22.00] | 14.90 [8.50, 22.00] | 0.760 |
| CK(U/L) | 234.00 [91.00, 690.50] | 276.00 [93.00, 857.00] | 0.414 |
| CK-MB(U/L) | 23.00 [12.00, 55.00] | 25.00 [14.00, 84.00] | 0.174 |
| Glucose(mmol/L) | 12.90 [9.20, 17.05] | 11.50 [8.10, 16.40] | 0.046 |
| Lactate(mmol/L) | 1.90 [1.30, 3.10] | 2.20 [1.50, 3.40] | 0.027 |

ALT: Alanine aminotransferase; APPT: Activated partial thrombin time; AST: Aspartate aminotransferase; BUN: blood urea nitrogen; CK: Creatine Kinase; CK-MB: Creatine kinase myocardial band; CRP: C-reactive protein; INR: International Normalized Ratio; PCT: Procalcitonin; PT: Prothrombin time; WBC: White blood cell; p < 0.05, statistically significant.

| **Supplementary Material 2** Baseline data of sepsis patients and ARDS after propensity matching in Tianjin Medical University General Hospital | | | |
| --- | --- | --- | --- |
| **Characteristic** | **Non-pulmonary infections and ARDS(n=387)** | **Pulmonary infections and ARDS(n=387)** | ***P*** |
| **Indicators of infection** | | | |
| WBCx10ˆ9/L | 13.74 [9.66, 19.21] | 11.12 [7.75, 18.62] | 0.001 |
| CRP(mg/L) | 9.68 [4.04, 20.00] | 11.60 [4.85, 20.00] | 0.356 |
| PCT(ng/mL) | 2.16 [0.75, 7.41] | 2.16 [0.89, 6.43] | 0.961 |
| **Physiology** | | | |
| Temperature, °C | 37.00 [36.50, 38.30] | 37.00 [36.50, 38.20] | 0.033 |
| Heart rate, beats per minute | 103.00 [87.00, 120.50] | 100.00 [82.00, 116.00] | 0.047 |
| Systolicblood pressure, mmHg | 115.00 [96.00, 135.00] | 127.00 [108.00, 153.00] | <0.001 |
| Diastolicblood pressure, mmHg | 60.00 [49.00, 75.00] | 63.00 [54.00, 76.00] | 0.011 |
| **Laboratory indicators** | | | |
| Platelet (×10ˆ9 /L) | 123.00 [62.50, 189.00] | 148.00 [74.00, 207.00] | 0.001 |
| Hemoglobin(g/dL) | 82.00 [66.00, 105.50] | 87.00 [70.00, 119.00] | <0.001 |
| PT(sec) | 13.20 [11.75, 15.00] | 13.10 [11.70, 14.70] | 0.989 |
| INR | 1.22 [1.08, 1.37] | 1.21 [1.09, 1.35] | 0.879 |
| APTT(sec) | 31.00 [28.10, 34.40] | 31.20 [28.40, 36.50] | 0.035 |
| ALT(U/L) | 29.00 [17.00, 60.00] | 34.00 [22.00, 69.00] | 0.013 |
| AST(U/L) | 44.00 [29.00, 97.50] | 54.00 [35.00, 103.00] | 0.025 |
| Albumin(g/L) | 31.00 [29.00, 33.00] | 31.00 [28.00, 35.00] | 0.280 |
| Creatinine(umol/L) | 163.00 [76.00, 267.50] | 121.00 [69.00, 267.00] | 0.029 |
| Bun (mmol/L) | 15.00 [9.00, 22.05] | 13.00 [8.00, 20.40] | 0.043 |
| CK(U/L) | 234.00 [88.50, 690.50] | 244.00 [86.00, 761.00] | 0.855 |
| CK-MB(U/L) | 23.00 [12.00, 54.00] | 22.00 [11.00, 89.00] | 0.797 |
| Glucose(mmol/L) | 12.90 [9.20, 17.05] | 10.40 [7.70, 15.80] | <0.001 |
| Lactate(mmol/L) | 1.90 [1.30, 3.10] | 2.00 [1.40, 2.80] | 0.701 |

ALT: Alanine aminotransferase; APPT: Activated partial thrombin time; AST: Aspartate aminotransferase; BUN: blood urea nitrogen; CK: Creatine Kinase; CK-MB: Creatine kinase myocardial band; CRP: C-reactive protein; INR: International Normalized Ratio; PCT: Procalcitonin; PT: Prothrombin time; WBC: White blood cell; *p* < 0.05, statistically significant.

**Supplementary Material 3**  Propensity match score in Tianjin Medical University General Hospital


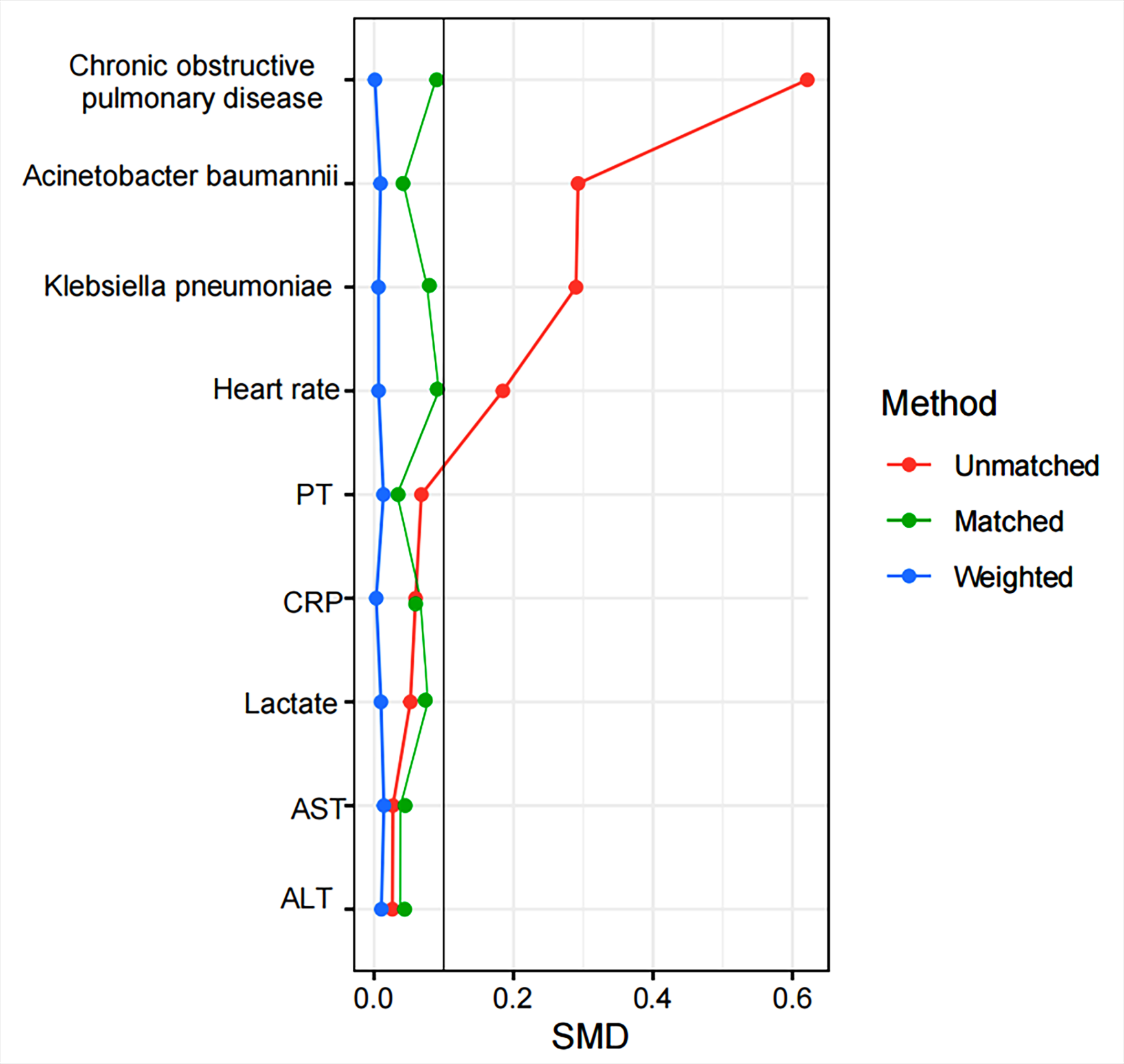


SMD<10% indicates that the propensity match score is very well. The matched variables are the metrics *P* < 0.05 in the original cohort of Table1, including all variables in the graph. ALT: Alanine aminotransferase; AST: Aspartate aminotransferase;AST: Aspartate aminotransferase;PT: Prothrombin time.

| **Supplementary Material 4** Baseline data of sepsis patients and ARDS in MIMIC IV database | | | |
| --- | --- | --- | --- |
| **Characteristic** | **Pulmonary infections and ARDS(n=172)** | **Non-pulmonary infections and ARDS(n=850)** | ***P*** |
| Age, years | 62.00 [51.75, 72.00] | 65.00 [56.00, 73.00] | 0.008 |
| Male sex, n (%) | 113 (65.7) | 585 ( 68.8) | 0.475 |
| **Co-morbid conditions, n (%)** | | | |
| Hypertension | 81 (47.1) | 436 ( 51.3) | 0.357 |
| Diabetes | 46 (26.7) | 228 ( 26.8) | 1.000 |
| Chronic obstructive pulmonary disease | 52 (30.2) | 122 ( 14.4) | <0.001 |
| Cardiovascular | 25 (14.5) | 142 ( 16.7) | 0.556 |
| Hepatic | 19 (11.0) | 52 ( 6.1) | 0.031 |
| Chronic kidney diseae | 7 ( 4.1) | 75 ( 8.8) | 0.052 |
| **Site of infection, n (%)** | | | |
| Lung | 172 ( 0.0) | 0 (0.0) | <0.001 |
| Abdominal | 0 ( 0) | 338( 39.76) | <0.001 |
| Urinary | 0 ( 0) | 128( 15.1) | <0.001 |
| Skin softtissue | 0 ( 0) | 84( 9.88) | <0.001 |
| Catheter | 0 ( 0) | 38( 4.47) | <0.001 |
| **Pathogenic microorganisms, n (%)** | | | |
| Acinetobacter baumannii | 48 ( 27.8) | 61 ( 7.2) | <0.001 |
| Klebsiella pneumoniae | 22 ( 12.79) | 8 ( 0.9) | <0.001 |
| Pseudomonas aeruginosa | 2 ( 1.2) | 5 ( 0.6) | 0.744 |
| Escherichiacoli | 10 ( 5.8) | 76 ( 8.9) | 0.231 |
| Staphylococcus aureus | 18 (10.5) | 84 ( 9.9) | 0.926 |
| **Respiration** | | | |
| SpO_2_, % | 93.00 [91.00, 95.00] | 94.00 [92.00, 95.00] | 0.006 |
| PaO_2_, mmHg | 111.00 [88.00, 167.00] | 118.00 [101.00, 168.00] | 0.004 |
| PaCO_2_, mmHg | 40.00 [36.75, 45.00] | 41.00 [36.00, 45.00] | 0.751 |
| FiO_2_, % | 100.00 [50.00, 100.00] | 100.00 [60.00, 100.00] | 0.003 |
| PaO_2_/FiO_2_ ratio | 166.00 [117.00, 220.50] | 161.00 [115.00, 211.48] | 0.520 |
| SpO_2_/FiO_2_ ratio | 166.67 [112.43, 192.00] | 172.73 [131.43, 230.00] | 0.003 |
| ROX | 6.45 [4.33, 8.24] | 6.00 [4.40, 8.25] | 0.793 |
| PEEP, cmH_2_O | 5.00 [5.00, 5.00] | 5.00 [5.00, 5.00] | 0.817 |
| **Indicators of infection** | | | |
| WBCx10ˆ9/L | 14.90 [11.38, 18.90] | 14.40 [11.70, 18.90] | 0.642 |
| **Physiology** | | | |
| Temperature,°C | 37.40 [37.10, 37.50] | 36.80 [36.60, 36.90] | <0.001 |
| Heart rate, beats per minute | 107.50 [95.00, 117.00] | 99.00 [89.25, 110.00] | <0.001 |
| Systolicblood pressure, mmHg | 90.00 [81.50, 97.00] | 87.00 [79.25, 95.00] | 0.032 |
| Diastolicblood pressure, mmHg | 47.00 [41.00, 52.00] | 45.00 [40.00, 49.75] | 0.015 |
| **Laboratory indicators** | | | |
| Platelet×10^^9^ /L | 149.50 [109.75, 198.25] | 139.50 [109.00, 189.00] | 0.177 |
| Hemoglobin(g/dL) | 10.10 [8.90, 11.40] | 9.70 [8.50, 10.80] | 0.005 |
| PT(s) | 14.60 [13.00, 16.42] | 15.00 [13.60, 16.60] | 0.03 |
| INR | 1.30 [1.20, 1.50] | 1.30 [1.20, 1.50] | 0.026 |
| APTT(s) | 31.05 [27.03, 38.75] | 33.10 [28.80, 41.18] | 0.008 |
| Creatinine(mg/dL) | 0.90 [0.70, 1.12] | 0.90 [0.80, 1.10] | 0.948 |
| Bun(mg/dL) | 16.00 [12.00, 21.00] | 17.00 [13.00, 23.00] | 0.01 |
| Glucose(mg/dL) | 126.75 [116.95, 148.10] | 127.60 [118.50, 140.50] | 0.74 |
| Lac (mmol/L) | 2.40 [1.70, 3.20] | 2.50 [1.80, 3.20] | 0.341 |
| **0utcome** | | | |
| ICU length of stay (days) | 2.21 [1.35, 3.42] | 1.90 [1.26, 2.96] | 0.001 |
| Inotropic/vasopressor support, n (%) | 12 ( 7.0) | 56 ( 6.6) | 0.985 |
| Renal replacement therapy, n (%) | 56 (32.6) | 150 ( 17.6) | <0.001 |
| SAPS II | 34.50 [26.00, 42.25] | 34.00 [27.00, 42.00] | 0.762 |
| SOFA | 5.00 [3.00, 7.00] | 5.00 [3.00, 6.00] | 0.442 |

APPT: Activated partial thrombin time; BUN: blood urea nitrogen; FiO_2_: Fraction of inspiration O_2_; INR: International Normalized Ratio; Lac: Lactates; PaCO_2_: partial pressure of carbon dioxide; PaO_2_: partial pressure of oxygen; PEEP: positive end-expiratory pressure; PT: Prothrombin time; ROX: Ratio of SpO_2_/FiO_2_ to respiratory rate; SAPSII: simplified acute physiology score; SOFA: Sequential organ failure assessment; SpO_2_: arterial oxygen saturation; WBC: White blood cell; *P* < 0.05, statistically significant.

| **Supplementary Material 5** Baseline data of sepsis patients and ARDS of eICU database | | | |
| --- | --- | --- | --- |
| **Characteristic** | **Pulmonary infections and ARDS(n=271)** | **Non-Pulmonary infections and ARDS(n=636)** | ***P*** |
| Age, years | 63.00 [53.00, 76.00] | 67.50 [56.00, 78.00] | 0.011 |
| Male sex, n (%) | 154 (56.8) | 355 ( 55.8) | 0.836 |
| **Co-morbid conditions, n (%)** | | | |
| Hypertension | 72 (26.6) | 122 ( 19.2) | 0.017 |
| Diabetes | 62 (22.9) | 113 ( 17.8) | 0.090 |
| Chronic obstructive pulmonary disease | 59 (21.8) | 80 ( 12.6) | 0.001 |
| Cardiovascular | 36 (13.3) | 76 ( 11.9) | 0.654 |
| Hepatic | 19 ( 7.0) | 30 ( 4.7) | 0.216 |
| Chronic kidney diseae | 34 (12.5) | 64 ( 10.1) | 0.324 |
| **Site of infection, n (%)** | | | |
| Lung | 271 ( 0.0) | 0 (0.0) | <0.001 |
| Abdominal | 0( 0) | 237( 37.26) | <0.001 |
| Urinary | 0( 0) | 94( 14.78) | <0.001 |
| Skin softtissue | 0( 0) | 65( 10.22) | <0.001 |
| Catheter | 0( 0) | 15( 2.36) | <0.001 |
| **Pathogenic microorganisms, n (%)** | | | |
| Acinetobacter baumannii | 64 (23.6) | 85 ( 13.4) | <0.001 |
| Klebsiella pneumoniae | 25 ( 9.22) | 5 ( 0.8) | <0.001 |
| Pseudomonas aeruginosa | 0 ( 0.0) | 2 ( 0.3) | 0.880 |
| Escherichiacoli | 24 ( 8.9) | 53 ( 8.3) | 0.898 |
| Staphylococcus aureus | 22 ( 8.1) | 36 ( 5.7) | 0.216 |
| **Respiration** | | | |
| SpO_2_, % | 93.00 [92.00, 96.00] | 93.00 [92.00, 96.00] | 0.362 |
| PaO_2_,mmHg | 83.00 [67.55, 108.00] | 85.00 [67.00, 109.00] | 0.596 |
| PaCO_2_, mmHg | 44.20 [36.75, 56.00] | 43.25 [36.00, 55.55] | 0.527 |
| FiO_2_, % | 55.00 [50.00, 85.00] | 55.00 [40.00, 70.00] | 0.005 |
| PaO_2_/FiO_2_ ratio | 155.00 [106.50, 210.00] | 167.00 [123.75, 221.25] | 0.009 |
| SpO_2_/FiO_2_ ratio | 166.67 [112.43, 192.00] | 172.73 [131.43, 230.00] | 0.003 |
| ROX | 8.50 [6.75, 11.00] | 8.90 [7.00, 12.30] | 0.003 |
| PEEP, cmH2O | 6.00 [5.00, 8.00] | 6.00 [5.00, 8.00] | 0.006 |
| **Indicators of infection** | | | |
| WBCx10ˆ9/L | 15.30 [10.40, 19.95] | 15.80 [10.80, 21.20] | 0.271 |
| Physiology | | | |
| Temperature, °C | 36.90 [36.60, 37.30] | 36.90 [36.60, 37.20] | 0.312 |
| Heart rate, beats per minute | 102.00 [90.50, 118.00] | 101.50 [91.00, 113.00] | 0.196 |
| Systolicblood pressure, mmHg | 168.00 [150.00, 191.50] | 164.00 [146.00, 187.00] | 0.095 |
| Diastolicblood pressure, mmHg | 86.00 [74.50, 99.00] | 85.00 [73.00, 96.00] | 0.219 |
| **Laboratory indicators** | | | |
| Platelet×10^^9^ /L | 167.00 [114.50, 223.00] | 186.00 [129.00, 254.25] | 0.001 |
| Hemoglobin(g/dL) | 10.10 [8.60, 11.90] | 10.10 [8.70, 11.60] | 0.796 |
| PT(s) | 16.00 [12.55, 17.50] | 17.50 [12.90, 17.50] | 0.3 |
| INR | 1.40 [1.10, 1.60] | 1.50 [1.10, 1.60] | 0.537 |
| APTT(s) | 40.60 [30.95, 40.60] | 40.60 [31.00, 40.60] | 0.897 |
| Creatinine(mg/dL) | 1.40 [0.88, 2.20] | 1.24 [0.81, 2.10] | 0.168 |
| Bun(mg/dL) | 29.00 [19.50, 41.00] | 29.00 [20.00, 45.00] | 0.716 |
| Glucose(mg/dL) | 172.00 [130.00, 228.50] | 167.50 [132.00, 226.00] | 0.84 |
| Lac (mmol/L) | 2.40 [1.60, 4.10] | 2.30 [1.60, 3.10] | 0.034 |
| **0utcome** | | | |
| ICU length of stay (days) | 5.62 [2.96, 9.23] | 4.17 [2.46, 7.97] | 0.01 |
| Inotropic/vasopressor support, n (%) | 109 (40.2) | 202 ( 31.8) | 0.017 |
| Renal replacement therapy, n (%) | 64 (23.6) | 153 ( 24.1) | 0.954 |
| SAPS II | 52.00 [30.00, 74.00] | 48.00 [30.00, 70.00] | 0.042 |
| SOFA | 8.00 [6.00, 11.00] | 8.00 [6.00, 10.00] | <0.001 |

APPT: Activated partial thrombin time; BUN: blood urea nitrogen; FiO_2_: Fraction of inspiration O_2_; INR: International Normalized Ratio; Lac: Lactates; PaCO_2_: partial pressure of carbon dioxide; PaO_2_: partial pressure of oxygen; PEEP: positive end-expiratory pressure; PT: Prothrombin time; ROX: Ratio of SpO_2_/FiO_2_ to respiratory rate; SAPSII: simplified acute physiology score; SOFA: Sequential organ failure assessment; SpO_2_: arterial oxygen saturation; WBC: White blood cell; *P* < 0.05, statistically significant.

| **Supplementary material 6** | Comparison of prognostic scores and oxygenation index between pulmonary infection group and non-pulmonary infection | | |
| --- | --- | --- | --- |
|  | Pulmonary infections and ARDS | Non-Pulmonary infections and ARDS | ***P*** |
| **MIMIC IV database** | | | |
| SOFA | 34.50 [26.00, 42.25] | 34.00 [27.00, 42.00] | 0.762 |
| SAPS II | 5.00 [3.00, 7.00] | 5.00 [3.00, 6.00] | 0.442 |
| ROX | 6.45 [4.33, 8.24] | 6.00 [4.40, 8.25] | 0.793 |
| PaO_2_/FiO_2_ | 166.00 [117.00, 220.50] | 161.00[115.00,211.48] | 0.520 |
| **eICU database** | | | |
| SOFA | 52.00 [30.00, 74.00] | 48.00 [30.00, 70.00] | 0.042 |
| SAPS II | 8.00 [6.00, 11.00] | 8.00 [6.00, 10.00] | <0.001 |
| ROX | 8.50 [6.75, 11.00] | 8.90 [7.00, 12.30] | 0.003 |
| PaO_2_/FiO_2_ | 155.00[106.50,210.00] | 167.00 [123.75, 221.25] | 0.009 |

FiO_2_: Fraction of inspiration O_2_; PaO_2_: partial pressure of oxygen;SAPSII: simplified acute physiology score; SOFA: Sequential organ failure assessment; *P* < 0.05, statistically significant.

**Supplementary material 7** KM curves of 90-day survival rate of sepsis patients and ARDS associated with pulmonary infection and non-pulmonary infection

**
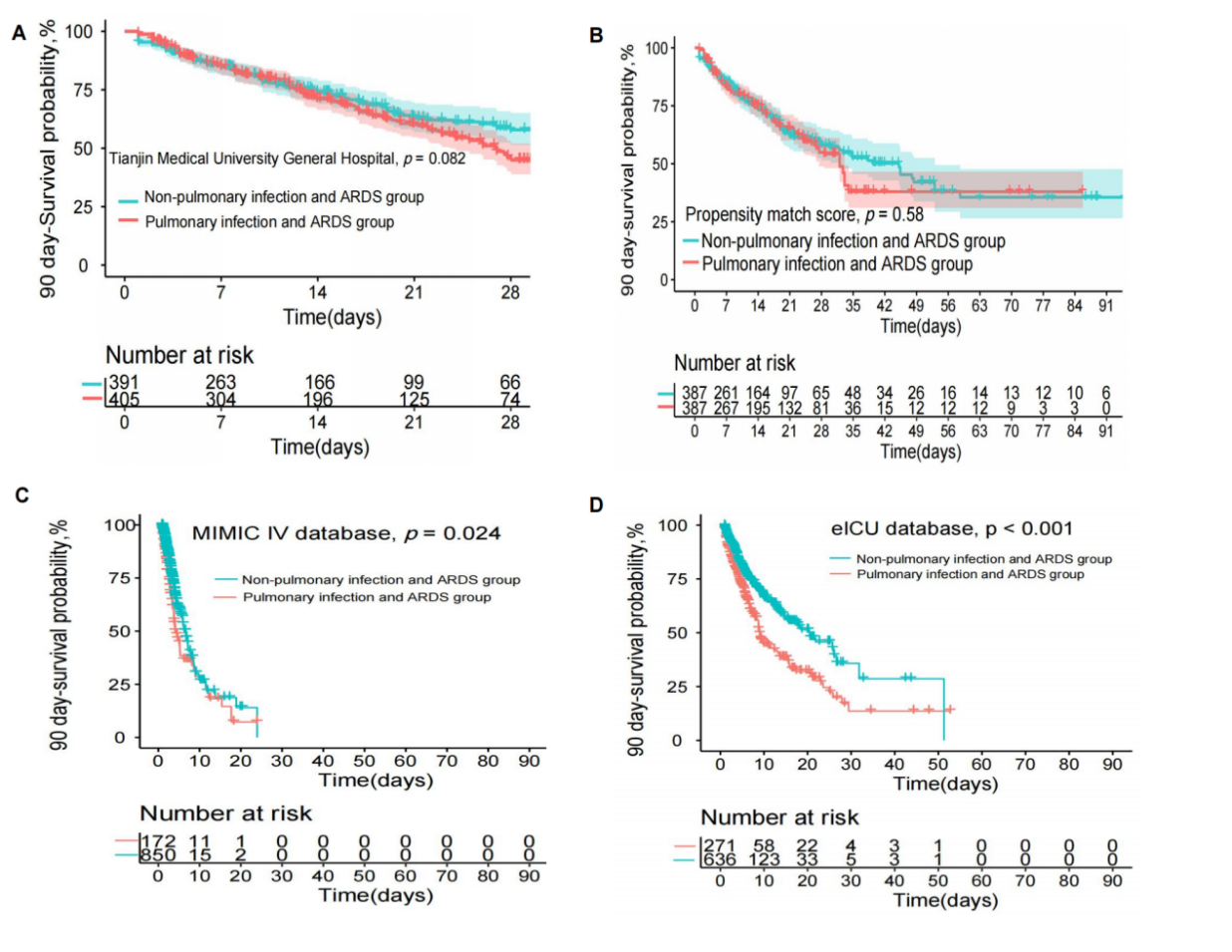
**

| **Supplementary material 8** | Correlation analysis between ROX and PaO_2_/FiO_2_ and SOFA and SAPS II | |
| --- | --- | --- |
|  | OR | *P* |
| **MIMIC IV database** | | |
| PaO_2_/FiO_2_ and SOFA | 0.052 | 0.095 |
| ROX and SOFA | 0.029 | 0.360 |
| PaO_2_/FiO_2_ and SPAS II | -0.011 | 0.730 |
| ROX and SPAS II | -0.022 | 0.490 |
| **eICU database** | | |
| PaO_2_/FiO_2_ and SOFA | -0.087 | 0.009 |
| ROX and SOFA | -0.098 | 0.003 |
| PaO_2_/FiO_2_ and SPAS II | 0.061 | 0.066 |
| ROX and SPAS II | 0.043 | 0.200 |

FiO_2_: Fraction of inspiration O_2_; PaO_2_: partial pressure of oxygen;SAPSII: simplified acute physiology score; SOFA: Sequential organ failure assessment; *P* < 0.05, statistically significant.

| **Supplementary Material 9** Subphenotypic analysis of COPD and non-COPD patients of ARDS patients with pulmonary infection | | | |
| --- | --- | --- | --- |
| **Characteristic** | **Non-COPD patients(n=230)** | **COPD patients(n=175)** | ***P*** |
| Age, years | 68.00 [55.00, 75.00] | 66.00 [55.50, 74.00] | 0.252 |
| Male sex, n (%) | 73 ( 31.7) | 65 ( 37.1) | 0.303 |
| **Co-morbid conditions, n (%)** | | | |
| Hypertension | 117 ( 50.9) | 101 ( 57.7) | 0.205 |
| Diabetes | 62 ( 27.0) | 56 ( 32.0) | 0.319 |
| Cardiovascular | 66 ( 28.7) | 49 ( 28.0) | 0.966 |
| Chronic obstructive pulmonary disease | 0 ( 0.0) | 175 (100.0) | <0.001 |
| Chronic kidney disease | 27 ( 11.7) | 53 ( 30.3) | <0.001 |
| Hepatic | 14 ( 6.1) | 28 ( 16.0) | 0.002 |
| **Pathogenic microorganisms** | | | |
| Acinetobacter baumannii | 59 ( 25.7) | 46 ( 26.3) | 0.976 |
| Klebsiella pneumoniae | 70 ( 30.4) | 57 ( 32.6) | 0.726 |
| Pseudomonas aeruginosa | 33 ( 14.3) | 19 ( 10.9) | 0.373 |
| Escherichiacoli | 17 ( 7.4) | 11 ( 6.3) | 0.813 |
| Staphylococcus aureus | 32 ( 13.9) | 17 ( 9.7) | 0.259 |
| **Indicators of infection** | | | |
| WBCx10ˆ9/L | 14.80 [9.62, 22.08] | 13.09 [8.37, 20.24] | 0.193 |
| CRP(mg/L) | 15.15 [6.96, 20.00] | 18.30 [6.50, 22.05] | 0.211 |
| PCT(ng/mL) | 2.44 [0.98, 8.43] | 3.22 [0.89, 16.22] | 0.149 |
| **Physiology** | | | |
| Temperature,°C | 37.40 [36.50, 38.30] | 37.30 [36.50, 38.60] | 0.607 |
| Heart rate, beats per minute | 112.00 [93.00, 128.00] | 110.00 [89.00, 125.00] | 0.247 |
| Systolicblood pressure, mmHg | 116.00 [90.50, 144.00] | 112.00 [91.00, 143.00] | 0.671 |
| Diastolicblood pressure, mmHg | 60.00 [48.00, 74.75] | 59.00 [48.00, 73.50] | 0.908 |
| **Laboratory indicators** | | | |
| Platelet (×10ˆ9 /L) | 132.00 [55.50, 201.50] | 96.00 [42.00, 185.50] | 0.086 |
| Hemoglobin(g/dL) | 85.00 [71.00, 115.75] | 78.00 [64.00, 104.50] | 0.005 |
| PT(sec) | 13.50 [12.10, 15.30] | 13.30 [12.20, 15.45] | 0.973 |
| INR | 1.24 [1.11, 1.41] | 1.23 [1.13, 1.41] | 0.981 |
| APTT(sec) | 31.10 [27.80, 37.18] | 31.90 [27.95, 36.85] | 0.697 |
| ALT(U/L) | 35.00 [22.00, 82.50] | 34.00 [21.00, 61.00] | 0.414 |
| AST(U/L) | 59.50 [35.25, 130.25] | 50.00 [32.00, 90.00] | 0.036 |
| Albumin(g/L) | 31.00 [27.00, 33.00] | 31.00 [27.00, 32.00] | 0.625 |
| Creatinine(umol/L) | 135.00 [77.25, 267.00] | 178.00 [82.50, 330.50] | 0.153 |
| Bun (mmol/L) | 14.00 [8.00, 20.00] | 15.50 [9.00, 23.00] | 0.076 |
| CK(U/L) | 255.00 [84.25, 851.25] | 315.00 [105.50, 858.00] | 0.282 |
| CK-MB(U/L) | 26.00 [14.00, 90.75] | 24.00 [11.50, 73.00] | 0.353 |
| Glucose(mmol/L) | 10.60 [7.55, 15.88] | 12.60 [8.90, 17.35] | 0.014 |
| Lactate(mmol/L) | 2.30 [1.60, 3.30] | 2.00 [1.40, 3.45] | 0.059 |
| **Outcome** | | | |
| ICU length of stay (days) | 13.19 [5.78, 23.46] | 13.59 [8.00, 25.50] | 0.465 |
| Inotropic/vasopressor support, n (%) | 136 ( 59.1) | 126 ( 72.0) | 0.01 |
| Renal replacement therapy, n (%) | 96 ( 41.7) | 72 ( 41.1) | 0.985 |
| APACHE II | 24.00 [18.25, 25.00] | 21.00 [15.00, 25.00] | 0.011 |
| SOFA | 9.00 [8.00, 12.00] | 9.00 [7.00, 12.00] | 0.155 |
| 28-day mortality | 122 ( 53.0) | 41 ( 23.4) | <0.001 |
| 90-day mortality | 125 ( 54.3) | 46 ( 26.3) | <0.001 |
| **Oxygenation indicators** | | | |
| FiO_2_ | 0.66 [0.50, 1.00] | 0.61 [0.50, 1.00] | 0.195 |
| PaO_2_,mmHg | 84.55 [67.73, 113.00] | 88.62 [67.85, 117.96] | 0.611 |
| SpO_2_,% | 92.00 [90.00, 95.00] | 93.00 [90.00, 96.00] | 0.13 |
| PaO_2_/FiO_2_ | 142.75 [85.65, 205.26] | 142.83 [92.60, 215.56] | 0.288 |
| ROX | 5.11 [3.69, 7.72] | 6.00 [4.10, 8.12] | 0.032 |
| Duration_mechanical_  ventilation | 10.00 [4.26, 16.34] | 8.53 [4.00, 16.00] | 0.527 |

| **Supplementary Material 10** Subphenotypic analysis of viral and non-viral patients of ARDS patients with pulmonary infection | | | |
| --- | --- | --- | --- |
| **Characteristic** | **Non-viral patients(n=333)** | **viral patients(n=72)** | ***P*** |
| Age, years | 66.00 [55.00, 74.00] | 71.50 [61.50, 76.00] | 0.048 |
| Male sex, n (%) | 115 ( 34.5) | 23 ( 31.9) | 0.777 |
| **Co-morbid conditions, n (%)** | | | |
| Hypertension | 178 ( 53.5) | 40 ( 55.6) | 0.846 |
| Diabetes | 102 ( 30.6) | 16 ( 22.2) | 0.200 |
| Cardiovascular | 94 ( 28.2) | 21 ( 29.2) | 0.987 |
| Chronic obstructive pulmonary disease | 141 ( 42.3) | 34 ( 47.2) | 0.531 |
| Chronic kidney disease | 66 ( 19.8) | 14 ( 19.4) | 1.000 |
| Hepatic | 35 ( 10.5) | 7 ( 9.7) | 1.000 |
| **Pathogenic microorganisms** | | | |
| Acinetobacter baumannii | 84 ( 25.2) | 21 ( 29.2) | 0.587 |
| Klebsiella pneumoniae | 105 ( 31.5) | 22 ( 30.6) | 0.983 |
| Pseudomonas aeruginosa | 43 ( 12.9) | 9 ( 12.5) | 1.000 |
| Escherichiacoli | 22 ( 6.6) | 6 ( 8.3) | 0.789 |
| Staphylococcus aureus | 38 ( 11.4) | 11 ( 15.3) | 0.476 |
| **Indicators of infection** | | | |
| WBCx10ˆ9/L | 14.53 [9.31, 21.54] | 12.06 [7.32, 17.75] | 0.026 |
| CRP(mg/L) | 15.20 [6.68, 20.00] | 19.80 [9.53, 20.00] | 0.220 |
| PCT(ng/mL) | 2.97 [0.97, 11.02] | 3.04 [0.89, 9.30] | 0.939 |
| **Physiology** | | | |
| Temperature,°C | 37.50 [36.50, 38.50] | 36.75 [36.50, 38.12] | 0.104 |
| Heart rate, beats per minute | 112.00 [92.00, 127.00] | 110.00 [88.75, 127.00] | 0.587 |
| Systolicblood pressure, mmHg | 115.00 [90.00, 143.00] | 116.50 [95.25, 151.25] | 0.529 |
| Diastolicblood pressure, mmHg | 60.00 [48.00, 73.00] | 59.50 [49.75, 76.50] | 0.480 |
| **Laboratory indicators** | | | |
| Platelet (×10ˆ9 /L) | 121.00 [47.00, 198.00] | 130.00 [50.50, 194.50] | 0.796 |
| Hemoglobin(g/dL) | 81.00 [67.00, 112.00] | 84.50 [69.75, 102.25] | 0.832 |
| PT(sec) | 13.50 [12.10, 15.70] | 13.05 [12.20, 15.03] | 0.627 |
| INR | 1.24 [1.11, 1.43] | 1.23 [1.13, 1.37] | 0.732 |
| APTT(sec) | 31.40 [27.80, 37.20] | 31.20 [28.37, 36.10] | 0.992 |
| ALT(U/L) | 35.00 [21.00, 77.00] | 33.50 [22.75, 61.00] | 0.733 |
| AST(U/L) | 55.00 [34.00, 112.00] | 55.00 [33.00, 81.00] | 0.357 |
| Albumin(g/L) | 31.00 [27.00, 33.00] | 30.00 [26.00, 32.00] | 0.109 |
| Creatinine(umol/L) | 159.00 [83.00, 286.00] | 114.00 [64.00, 319.00] | 0.068 |
| Bun (mmol/L) | 15.00 [9.00, 22.00] | 13.50 [6.95, 21.12] | 0.119 |
| CK(U/L) | 285.00 [101.00, 856.00] | 222.00 [79.00, 883.25] | 0.36 |
| CK-MB(U/L) | 25.00 [13.00, 88.00] | 28.00 [15.50, 66.00] | 0.791 |
| Glucose(mmol/L) | 11.80 [8.20, 16.50] | 10.15 [7.77, 15.90] | 0.173 |
| Lactate(mmol/L) | 2.20 [1.50, 3.50] | 2.25 [1.58, 2.92] | 0.879 |
| **Outcome** | | | |
| ICU length of stay (days) | 13.00 [6.00, 22.83] | 18.50 [9.81, 30.58] | <0.001 |
| Inotropic/vasopressor support, n (%) | 216 ( 64.9) | 46 ( 63.9) | 0.983 |
| Renal replacement therapy, n (%) | 137 ( 41.1) | 31 ( 43.1) | 0.867 |
| APACHE II | 22.00 [16.00, 25.00] | 23.50 [19.00, 26.00] | 0.016 |
| SOFA | 8.00 [7.00, 12.00] | 11.00 [8.75, 13.00] | <0.001 |
| 28-day mortality | 133 ( 39.9) | 30 ( 41.7) | 0.890 |
| 90-day mortality | 139 ( 41.7) | 32 ( 44.4) | 0.772 |
| **Oxygenation indicators** | | | |
| FiO_2_ | 0.61 [0.50, 1.00] | 0.61 [0.50, 1.00] | 0.975 |
| PaO_2_,mmHg | 85.30 [67.25, 117.80] | 89.30 [70.55, 111.00] | 0.977 |
| SpO_2_,% | 92.00 [90.00, 96.00] | 93.00 [90.00, 96.25] | 0.524 |
| PaO_2_/FiO_2_ | 141.67 [88.62, 208.20] | 145.38 [90.22, 202.33] | 0.988 |
| ROX | 5.59 [3.84, 7.95] | 5.71 [4.01, 8.28] | 0.606 |
| Duration_mechanical_ventilation | 8.25 [4.00, 15.35] | 13.47 [5.43, 23.01] | 0.001 |

| **Supplementary Material 11** Subphenotypic analysis of abdominal infectionand non-abdominal infection of ARDS patients with non-pulmonary infection | | | |
| --- | --- | --- | --- |
| **Characteristic** | **Non-viral patients(n=226)** | **viral patients(n=165)** | ***P*** |
| Age, years | 65.00 [54.00, 73.00] | 70.00 [60.00, 76.00] | 0.003 |
| Male sex, n (%) | 90 ( 39.8) | 64 ( 38.8) | 0.919 |
| **Co-morbid conditions, n (%)** | | | |
| Hypertension | 125 ( 55.3) | 89 ( 53.9) | 0.868 |
| Diabetes | 67 ( 29.6) | 56 ( 33.9) | 0.428 |
| Cardiovascular | 86 ( 38.1) | 51 ( 30.9) | 0.175 |
| Chronic obstructive pulmonary disease | 31 ( 13.7) | 32 ( 19.4) | 0.171 |
| Chronic kidney disease | 58 ( 25.7) | 36 ( 21.8) | 0.448 |
| Hepatic | 27 ( 11.9) | 28 ( 17.0) | 0.206 |
| **Pathogenic microorganisms** | | | |
| Acinetobacter baumannii | 32 ( 14.2) | 24 ( 14.5) | 1.000 |
| Klebsiella pneumoniae | 34 ( 15.0) | 40 ( 24.2) | 0.031 |
| Pseudomonas aeruginosa | 22 ( 9.7) | 20 ( 12.1) | 0.557 |
| Escherichiacoli | 18 ( 8.0) | 17 ( 10.3) | 0.535 |
| Staphylococcus aureus | 22 ( 9.7) | 19 ( 11.5) | 0.689 |
| **Indicators of infection** | | | |
| WBCx10ˆ9/L | 13.77 [9.73, 19.36] | 13.74 [9.80, 18.51] | 0.947 |
| CRP(mg/L) | 10.35 [3.49, 20.00] | 8.68 [4.34, 20.00] | 0.75 |
| PCT(ng/mL) | 2.32 [0.85, 6.97] | 2.02 [0.70, 8.07] | 0.661 |
| **Physiology** | | | |
| Temperature,°C | 36.95 [36.50, 38.20] | 37.50 [36.50, 38.50] | 0.061 |
| Heart rate, beats per minute | 103.00 [90.00, 120.00] | 102.00 [85.00, 121.00] | 0.351 |
| Systolicblood pressure, mmHg | 117.00 [92.25, 135.00] | 114.00 [100.00, 134.00] | 0.985 |
| Diastolicblood pressure, mmHg | 61.50 [49.25, 75.00] | 59.00 [49.00, 74.00] | 0.473 |
| **Laboratory indicators** | | | |
| Platelet (×10ˆ9 /L) | 123.00 [61.25, 177.75] | 127.00 [67.00, 201.00] | 0.199 |
| Hemoglobin(g/dL) | 84.50 [65.25, 107.00] | 80.00 [67.00, 103.00] | 0.465 |
| PT(sec) | 13.20 [11.80, 15.28] | 13.10 [11.60, 14.70] | 0.563 |
| INR | 1.23 [1.09, 1.41] | 1.21 [1.09, 1.33] | 0.527 |
| APTT(sec) | 31.05 [28.10, 34.65] | 30.90 [28.00, 34.40] | 0.338 |
| ALT(U/L) | 34.00 [17.25, 68.75] | 27.00 [17.00, 57.00] | 0.093 |
| AST(U/L) | 45.00 [30.00, 115.25] | 42.00 [27.00, 95.00] | 0.199 |
| Albumin(g/L) | 31.00 [28.25, 33.00] | 31.00 [30.00, 32.00] | 0.601 |
| Creatinine(umol/L) | 164.50 [79.25, 304.25] | 159.00 [72.00, 267.00] | 0.419 |
| Bun (mmol/L) | 15.00 [9.00, 22.42] | 15.00 [9.00, 21.90] | 0.918 |
| CK(U/L) | 283.00 [114.50, 772.25] | 189.00 [67.00, 531.00] | 0.007 |
| CK-MB(U/L) | 28.00 [14.00, 68.00] | 20.00 [12.00, 39.00] | 0.005 |
| Glucose(mmol/L) | 12.90 [9.12, 16.67] | 13.20 [9.20, 17.40] | 0.623 |
| Lactate(mmol/L) | 2.15 [1.40, 3.70] | 1.70 [1.30, 2.50] | 0.001 |
| **Outcome** | | | |
| ICU length of stay (days) | 11.80 [5.00, 22.00] | 10.44 [5.74, 19.00] | 0.576 |
| Inotropic/vasopressor support, n (%) | 143 ( 63.3) | 99 ( 60.0) | 0.58 |
| Renal replacement therapy, n (%) | 80 ( 35.4) | 39 ( 23.6) | 0.017 |
| APACHE II | 17.00 [13.00, 23.00] | 15.00 [13.00, 22.00] | 0.153 |
| SOFA | 8.00 [5.00, 10.00] | 7.00 [5.00, 9.00] | 0.141 |
| 28-day mortality | 63 ( 27.9) | 45 ( 27.3) | 0.986 |
| 90-day mortality | 75 ( 33.2) | 50 ( 30.3) | 0.621 |
| **Oxygenation indicators** | | | |
| FiO_2_ | 0.53 [0.50, 0.80] | 0.60 [0.50, 0.80] | 0.847 |
| PaO_2_,mmHg | 103.85 [79.25, 130.74] | 110.13 [87.94, 140.50] | 0.030 |
| SpO_2_,% | 95.00 [92.00, 97.00] | 96.00 [92.00, 98.00] | 0.137 |
| PaO_2_/FiO_2_ | 189.42 [128.72, 225.71] | 202.14 [145.86, 249.16] | 0.016 |
| ROX | 6.79 [4.67, 9.90] | 6.64 [4.67, 8.82] | 0.692 |
| Duration_mechanical_ventilation | 6.00 [2.30, 12.00] | 7.00 [3.00, 14.47] | 0.364 |
